# Insights of Oncofertility in Mexico and Latin America

**DOI:** 10.3389/fonc.2022.811464

**Published:** 2022-05-06

**Authors:** Francisco Jiménez-Trejo, Cristian Arriaga-Canon, Luis A. Herrera, Telma Lisboa-Nascimento, Daniel Diaz

**Affiliations:** ^1^ Instituto Nacional de Pediatría, Ciudad de México, Mexico; ^2^ Instituto Nacional de Cancerología, Ciudad de México, Mexico; ^3^ Instituto Nacional de Medicina Genómica, Ciudad de México, Mexico; ^4^ Universidade Federal de São Paulo (UNIFESP, EPM), Sao Paulo, Brazil; ^5^ Centro de Ciencias de la Complejidad, Universidad Nacional Autónoma de México, Ciudad de México, Mexico

**Keywords:** cancer survivors, cancer therapy, anticancer treatment, global community, oncology

## Introduction

Oncofertility is an interdisciplinary field placed at the intersection of oncology and reproductive medicine, which seeks to expand fertility options for cancer survivors (1). Pioneered by Dr. Jacques Donnéz (2004) work, this emerging branch of medicine has revolutionized the care of patients whose fertility may be threatened by anticancer treatments by protecting patients’ gonads in a systematic and timely manner (2). Although guidelines related to oncofertility have been in place for approximately 15 years in the United States, Europe, and Australia, Mexico has lagged on this front (just a few months ago a Mexican oncofertility consortium was founded; however, until now there has been little communication and dissemination among peers and those of us who wish to equalize the delay in the matter). Furthermore, there is little awareness in Mexico of international communication networks that provide information and resources on oncofertility, such as the Northwestern University Oncofertility Consortium, led by Dr. Teresa K. Woodruff, who first coined the term “oncofertility” (2006) and now, the recently created Global Community of Practice for Oncofertility (2020) (3–5).

According to the epidemiological transition that is taking place worldwide, it is expected that by 2030 low-income countries such as Mexico and the Latin American region will have about 70% of deaths from cancer; although in recent years, cancer survival rates have increased among children and young people thanks to increasingly effective cancer therapies and a wider variety of therapeutic options (6). However, this introduces the need for innovative techniques to preserve fertility in cancer survivors, both male, and female whose reproductive years still lie ahead (children, adolescents, and adults of both sexes). Currently, better techniques exist for preserving fertility and greater knowledge of the options for its preservation, emerging options to preserve the growth and fertility processes of the patients’ gonads that will undergo radiotherapy or chemotherapy in both sexes. An integral part of oncofertility is close, personalized communication between doctors, patients, and patients’ families (7). This occurs in conjunction with psychological support and medical care, in addition to scientific research in a systematic and timely manner.

## Commentary and Discussion

Several factors could explain Mexico’s underdeveloped oncofertility infrastructure. First, Mexico lacks the universal health coverage that is present in most developed countries. Furthermore, developing countries like Mexico do not have the same infrastructure tools or public health policy as developed countries, in addition to lacking an adequate number of physicians, psychologists, and researchers who specialize in reproductive biology. Together, these factors translate into a lack of infrastructure to support young cancer patients to preserve their long-term fertility into adult life.

Mexico needs a futuristic vision and continued leadership in the field of oncofertility. As Dr. Jacques Donnéz said, “We need to protect human fertility in cancer patients before the administration of any antitumor treatment that has the potential to affect germ cells. This will protect the functionality of the gonads in boys and girls exposed to radiotherapy and chemotherapy so that they can conceive children in the future if they wish”.

We believe that it is necessary to establish a National Oncofertility Service and an Experimental Oncofertility Laboratory in Mexico, sponsored by the government, the private sector, and non-governmental organizations to seek institutional agreements to make this project grow in favor of Mexican children and for other Latin American countries. Brazil (Brazilian Oncofertility Consortium/Rede Brasileira de Oncofertilidade), Peru (Peruvian Oncofertility Network), and Panama are now part of the Global Community of Practice for Oncofertility and are more advanced in the matter than the rest of the Latin American countries. This is with aim of the following objectives:

1) Protect the health of the individual, emphasizing reproductive health and educating both the patient and his family about the potential reproductive implications of cancer. 2) Establish communication and provide personalized support to the patient’s family to expand medical perceptivity and ensure long-term follow-up of the patient. 3) Provide adequate and personalized psychological support to the patient and his family to avoid unnecessary emotional stress. 4) Establish an oncofertility research unit for basic, clinical, and translational research to develop better fertility preservation protocols. 5) Prioritize training and provide feedback from a new generation of highly specialized reproductive biology physician’ doctors, psychologists, nutritionists, and researchers. 6) Create an International Latino Oncofertility Consortium together with other Latin American countries, create networks of researchers and organize scientific congresses related to oncofertility, among other related pending issues (7, 8).

With these motivations in mind, it is imperative to focus on the creation of this site, in collaboration with the corresponding authorities. This site is crucial for the long-term reproductive health of pediatric cancer patients in our National Institute of Pediatrics and other hospitals, and in the short term, it would position Mexico, Brazil, Argentina, Peru, and Panama as some of the leaders of this emerging interdisciplinary field in Latin America (8, 9). The nature of oncofertility, bridging reproductive health and oncology, basic science and clinical research, medical and social science, and the academy and the public (work relies on collaboration). The collective knowledge and experiences of the international network are what drive the success of the global oncofertility effort, an imperative approach to the Latin American countries that are lagging far behind (5).

**Figure 1:**
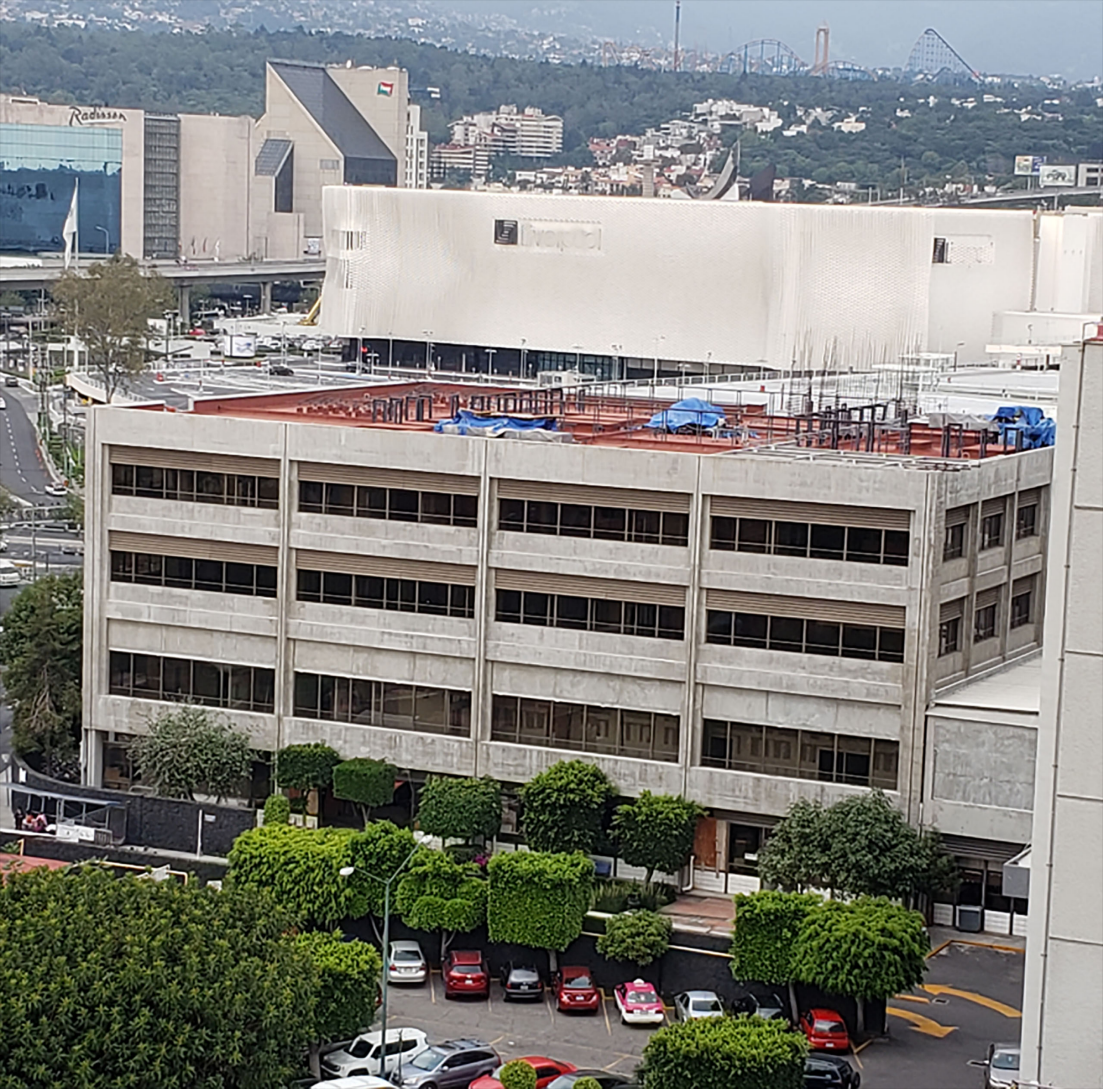
The image shows the building under construction of the new Hemato-Oncology Unit and the probable site of operation of the Oncofertility Service at the National Pediatric Institute of Mexico.

Finally, with the consent of Dr. Teresa K. Woodruff and Lauren M. Ataman, we seek to initiate an oncofertility program in Mexico to adhere to all these fundamental precepts together with the Global Community of Oncofertility practice, which has become essential for those of us who are attentive in supporting these pediatric patients and their families mainly, and in this way, avoid the delay in this issue of emergent medicine (**Figure 1**).

## Author Contributions

All authors listed have made a substantial, direct, and intellectual contribution to the work and approved it for publication.

## Conflict of Interest

The authors declare that the research was conducted in the absence of any commercial or financial relationships that could be construed as a potential conflict of interest.

## Publisher’s Note

All claims expressed in this article are solely those of the authors and do not necessarily represent those of their affiliated organizations, or those of the publisher, the editors and the reviewers. Any product that may be evaluated in this article, or claim that may be made by its manufacturer, is not guaranteed or endorsed by the publisher.
